# From Acute to Chronic Tinnitus: Pilot Data on Predictors and Progression

**DOI:** 10.3389/fneur.2020.00997

**Published:** 2020-09-11

**Authors:** Veronika Vielsmeier, Ryan Santiago Stiel, Pingling Kwok, Berthold Langguth, Martin Schecklmann

**Affiliations:** ^1^Department of Otorhinolaryngology, University of Regensburg, Regensburg, Germany; ^2^Department of Otorhinolaryngology, Klinikum St. Elisabeth Straubing GmbH, Straubing, Germany; ^3^Department of Psychiatry and Psychotherapy, University of Regensburg, Regensburg, Germany

**Keywords:** acute tinnitus, chronic tinnitus, questionnaire, causes for progression, predictors

## Abstract

Little is known about the transition from acute tinnitus to chronic tinnitus. By means of this study, we are attempting to close this gap by presenting prospective pilot data of patients with acute tinnitus, followed by tracking their condition's trajectory over a period of 6 months. Forty-nine patients presenting with acute tinnitus (duration < 28 days) were recruited in two clinics. We recorded demographic and clinical tinnitus-related data as well as data on personality, health, treatments, and life-style, during patients' first appearance in the clinic and again three and 6 months thereafter. Standard audiograms were performed at the first and the second visit. Nine (18.4%) patients showed full remission of their tinnitus. These patients differed from patients with a chronic course of tinnitus by shorter tinnitus duration, lower fear-related hyperacusis, higher proportion of female gender, increased ear pressure, and lower levels of alcohol consumption. Among the patients with a chronification of tinnitus, there was no change in tinnitus characteristics. However, their tinnitus distress improved moderately over time. These preliminary data are in line with earlier studies that have shown that only a small proportion of those patients presenting in the clinic with acute tinnitus experience a full remission. The results of this study can be of service in deducing hypotheses for the transition from acute to chronic tinnitus and in developing designs for interventional studies.

## Introduction

Subjective chronic tinnitus has a high prevalence, affecting 10–15% of adults worldwide (1, 2), and is responsible for high costs for health care systems (3). Research on chronic tinnitus has been growing during the last decade.

Several hypotheses about risk factors for developing chronic or decompensated tinnitus exist. Hearing impairment can be found in almost all patients with chronic tinnitus (4, 5). However, not every patient with hearing difficulty has tinnitus, meaning that hearing impairment may be a required but not sufficient risk factor. For example, Muhlmeier et al. published a study including 113 patients with acute idiopathic sudden sensorineural hearing loss. Here, tinnitus loudness decreased rapidly in cases of mild-moderate hearing loss and tinnitus had completely resolved in two-thirds of the patients after 3 months. In contrast, when tinnitus is associated with severe-profound hearing loss, it improved significantly less (6). Also, temporomandibular joint or neck pathologies are discussed as moderators for tinnitus (7). Additional comorbidities such as depression (8), anxiety (8), hyperacusis (9), and personality traits such as neuroticism (10), are discussed as determining factors for the development of decompensated tinnitus, resulting in patients seeking for help. Anxiety disorders in tinnitus patients seem to be associated with more psychosocial stress (11). Other studies could show that low resilience is associated with low emotional health and distressing tinnitus (12). D'Amelio et al. described a high correlation between the degree of depression on one side and the dysfunctional coping with stress and emotional distress caused by tinnitus on the other side, frequently present in acute tinnitus cases (13).

Most findings on risk factors for chronic or decompensated tinnitus are based on cross-sectional and retrospective datasets or specific populations. Up to now, prospective and longitudinal studies are sparse. Overall, the transition and progression from acute to chronic tinnitus has not been sufficiently addressed.

We were only able to identify two studies investigating the course of acute tinnitus. One study followed the trajectory of tinnitus over 6 months (14). There, 10 out of 44 patients showed full recovery. Tinnitus-attributed sleep disturbance, anxiety, and life satisfaction, each assessed at first investigation, independently predicted tinnitus distress at the second assessment 6 months later in patients with a chronic course of tinnitus. These three variables together predicted 56% of the variance of tinnitus distress at the time of the second assessment. This is in accordance with a follow-up study of patients with tinnitus after 2 years, showing that severe tinnitus suffering was associated with a higher likelihood of anxiety disorders, more psychosocial stress and lower global assessment of function scores (11).

The second observational study on the development of acute tinnitus over time showed that almost 90% of participants developed chronic tinnitus, only five out of 41 patients showed complete remission after 6 months (15). Aspects of mental health like level of depression and anxiety at the beginning of tinnitus seem to be predicting a higher level of tinnitus decompensation after 6 months (15).

The prevalence data of these two prospective studies are in contrast to a retrospective study by Forti et al. of 269 patients who were treated with Tinnitus Retraining Therapy and sound generators, 26% did not use the sound generator in a mean time of 49 (+-16,89) months of follow-up, because their tinnitus apparently was no longer a problem (16).

In sum, data on the progression of acute tinnitus are limited, and the focus seems to be on the prediction of tinnitus worsening and not on predictors for tinnitus remission; or studies are based on a specific sub-group of patients with sudden hearing loss. Thus, we performed a two-center observational survey to evaluate possible predictors for tinnitus remission and investigate the transition from acute to chronic tinnitus during a time interval of 6 months after onset.

## Materials and Methods

We included 50 patients presenting in either one of two clinics of Otorhinolaryngology in Eastern Bavaria (Regensburg, Straubing), beginning from October 2013, with a duration of acute tinnitus of up to 28 days. For this work, acute tinnitus was defined by a tinnitus duration below 28 days, with the intention of investigating the transition from acute to chronic tinnitus. There is heterogeneity of definition of acute (<3 months), sub-acute (<6 months), and chronic tinnitus (>6 months but also >3 months) in the literature (15, 17). However, as we were interested in the transition from one stage to the other, we restricted our inclusion criteria to the very acute duration of tinnitus and decided to conduct the follow-up visits 3 and 6 months after the first consultation.

All patients gave written informed consent to the study which was approved by the ethics committee of the University of Regensburg (13-101-0160).

All patients presented twice in the hospital—for the first consultation and a first follow-up 3 months thereafter. The first contact took place in emergency settings. Three months after the second visit, patients received questionnaires by mail. We recorded different categories of data (i.e., demographic, tinnitus-related, audiology, psychopathological, health-related, treatment and life-style data). Most of the data were ratings or subjective reported variables. Audiology was recorded by the investigator team (pure tone audiogram; frequencies 125 Hz, 250 Hz, 500 Hz, 1 kHz, 2 kHz, 3 kHz, 4 kHz, 6 kHz, and 8 kHz). Standardized ratings included the Tinnitus Sample Case History Questionnaire (18) for tinnitus characteristics, numeric ratings scales for different aspects of tinnitus (19), the Mini Tinnitus Questionnaire (20) for measurement of tinnitus distress, the Big-Five-Inventory-10 (21) for measurement of five personality traits, the Life Orientation Test (LOT-R) (22) for measurement of optimism and pessimism, and the Generalized Anxiety Disorder 7 (GAD-7) of the Patient Health Questionnaire (PHQ) (23).

For group comparisons of categorial variables we used chi-square tests of independence, and of metric variables Student *t*-tests. Longitudinal analyses were done with Student *t*-test for dependent samples or, in the case of three observations, with analyses of variance with the within-subjects factor time. Significance threshold was set to 5%. Analyses were done with SPSS 24 (IBM SPSS Statistics).

## Results

### Sample Description

Fifty patients presented from October 2013 to December 2016 with a tinnitus onset of up to 28 days. Due to a high number of missing values, one patient had to be excluded from the study. The analysis sample consisted of 49 patients presenting in either one of two clinics for Otorhinolaryngology in Eastern Bavaria (Regensburg: *n* = 14; Straubing: *n* = 35). The recruitment time interval was from October 2013 to December 2016.

### Demographics

Twenty-one out of these 49 patients were females. Mean age was 46 ± 14 years (range: 21–81 years). Seventeen reported A-levels or equivalently high education. Thirty-one were married, seven with and 11 without partnership. Mean height was 1.73 ± 0.76 m and mean weight was 79 ± 14 kg. The mean number of alcoholic drinks per week was 2.8 ± 3.6. Eight patients reported regular smoking. Mean hearing (dB HL) loss was 27 ± 29 on the right and 24 ± 22 on the left side.

### Tinnitus Characteristics

Tinnitus duration was 8.5 ± 5.3 days (range: 1–25 days), tinnitus loudness was 44 ± 25, tinnitus awareness 61 ± 31, and tinnitus annoyance 54 ± 34 (all three variables with a scale from 0 to 100). Numeric ratings showed values for severity of 3.2 ± 1.1 (on a scale from 0 to 5). Numeric ratings with a scale from 0 to 10 showed 4.3 ± 2.7 for loudness, 5.1 ± 3.0 for discomfort, 5.3 ± 3.2 for annoyance, 5.5 ± 3.4 for ignorability, and 5.2 ± 3.1 for unpleasantness. The Mini Tinnitus Questionnaire showed a mean value of 11.6 ± 6.5 (range 0–23) indicating moderate distress in average. Eighteen (37%) patients reported tinnitus in relatives, 12 reported a gradual and 37 a sudden beginning of tinnitus. All patients experienced non-pulsatile tinnitus. Nineteen (39%) reported fluctuations of their tinnitus over time. Twenty patients had tinnitus purely on the right, 18 purely on the left side. Three reported a right-dominant tinnitus, five a left-dominant, and three patients equal distributed tinnitus over both ears. Most patients reported a tonal tinnitus (*n* = 39), two noise-like, one crickets-like, and seven other sounds. With respect to pitch, tinnitus was rated very high in six, high in 24, middle in 14, and low in five patients. Thirty-one reported maskability of tinnitus by sounds, 10 patients reported it not maskable, and eight were unable to answer this question. Modulation of tinnitus by somatic maneuvers was possible in 16 (33%) patients.

### Comorbidities

Half of patients (*n* = 23; 46.9%) reported worsening of tinnitus by stress and the remaining patients reported no impact of stress on their tinnitus. Only one patient was equipped with hearing aids. Thirteen patients reported painful hyperacusis, 27 had no hyperacusis, and nine patients did not answer this question. Fear-related hyperacusis was 2.8 ± 1.0 on a scale from 1 to 5.

Headache was prevalent in 14 patients, temporomandibular joint problems in 11 patients, neck pain in 22 patients, and general pain in six patients. Two patients reported concomitant psychiatric treatment.

With respect to putative tinnitus-causing factors, five patients reported to have a noisy hobby, 29 reported stress in the last weeks, five an acute and two long lasting noise exposition. Ear infections in the previous weeks were present in three patients, a common cold in nine patients, a recent hearing loss in 18 and a long-lasting hearing loss in eight patients. Ear pressure was prevalent in 28 patients and vertigo in 12 patients. Two patients reported a diving accident, six patients ear surgery, one patient acute and six patients earlier cervical spine injury. One patient reported head injury in the last weeks before tinnitus onset and two in the past.

With respect to diseases, we asked for diabetes (*n* = 2), stroke (*n* = 1), heart attack (*n* = 1), hypertension (*n* = 16), hypotension (*n* = 2), diseases of the immune system (*n* = 4), high cholesterol (*n* = 11), and gout (*n* = 3). No patient had any kidney disease or an infectious disease.

Concerning regular medication, five patients took anticoagulants, four diuretics, 15 blood pressure medication, eight antibiotics, and three contraceptives. No patient was undergoing chemotherapy.

### Treatment

During the observation period 30 patients (61.2%) received tinnitus treatment. Nineteen received steroid hormones (cortisone, prednisolone, and dexamethasone), five local anesthetics (lidocaine, procaine, and novocaine), six antihistamines (betahistine, dimenhydrinate), eight cardiac medication (several different medications), and nine herbal drugs (different herbal drugs and vitamines). Eleven underwent physiotherapy, two psychosomatic interventions (rehabilitation, autogenic training), one hyperbaric oxygen therapy. No ear surgery was reported. Four patients could not specify the treatment they received.

### Psychological Measures

The evaluation of personality traits with the Big-Five Personality Inventory (scale from 1 to 5), revealed extraversion to be 3.4 ± 1.0, neuroticism 3.2 ± 0.9, openness 3.5 ± 0.9, conscientiousness 4.3 ± 0.6, and agreeableness 3.1 ± 0.8. Pessimism was 11.1 ± 2.2 and optimism 6.6 ± 2.4, both measured on a scale from 1 to 15. Depressivity on a scale from 0 to 2 was 0.7 ± 0.9, and anxiety on a scale from 0 to 15 was 6.5 ± 4.4.

### Predictor Analysis

Nine out of 49 patients (18.4%) showed a full remission of tinnitus after 3 and 6 months. All measured variables were tested for group differences. Patients with full remission differed from patients with “chronic tinnitus” with respect to tinnitus duration, fear-related hyperacusis, drinks per week, gender, and ear pressure (i.e., tinnitus remission was associated with the following characteristics at baseline: shorter tinnitus duration, lower fear-related hyperacusis, lower consumption of alcoholic drinks, higher incidence of increased ear pressure and a higher proportion of women). For statistical values see Table 1. All other variables turned out not to differ significantly between groups.

**Table 1:** Significant differences in patients with full remission vs. patients with chronic course of tinnitus.

	**Remitted tinnitus (*n* = 9)**	**Chronic tinnitus (*n* = 40)**	
Sex (female/male)	7/2	14/26	χ^2^ = 5.490; df = 1; *p* = 0.019
Tinnitus duration (days)	4.78 ± 12.74	9.38 ± 5.36	*T* = 2.501; df = 47; *p* = 0.016
Loudness hyperacusis (never/rarely/sometimes/ usually/always)	2.00 ± 1.00	2.98 ± 0.95	*T* = 2.764; df = 47; *p* = 0.008
Drinks per week (500 ml beer or 250 ml wine)	0.44 ± 0.88	3.33 ± 3.85	*T* = 2.217; df = 47; *p* = 0.032
Ear pressure (yes/no)	8/1	20/20	χ^2^ = 4.537; df = 1; *p* = 0.033

### Longitudinal Analysis

For 37 out of the 40 patients with chronic tinnitus complete data sets of both follow-up visits were available. These patients showed no significant changes over time for tinnitus loudness or tinnitus awareness, but for tinnitus annoyance, discomfort, ignorability, unpleasantness, and distress. The reduction of the Mini TQ score over the observation period of 6 months was in the range of a medium effect size (Cohen's d(?) 0.691). Tinnitus laterality, type, and subjective reported frequency were similar for both visits as indicated by (near) significant chi-square tests. For details see Table 2. Hearing functions improved from visit 1 to visit 2, but there was no significant correlation between changes in hearing and changes in annoyance, discomfort, ignorability, unpleasantness, and distress. Correlations analyses were not significant (all *r* < 0.296; all *p* > 0.094).

**Table 2:** Course of tinnitus related variables over time in patients with chronic tinnitus.

	**Visit 1**	**Visit 2 (3 months)**	**Visit 3 (6 months)**	**Statistics**
Tinnitus loudness (0–100)	46.6 ± 25.0	43.7 ± 25.7	46.5 ± 23.7	*F* = 0.545; df = 2,70; *p* = 0.582
Tinnitus loudness (0–10)	4.6 ± 2.9	3.9 ± 2.9	3.9 ± 2.9	*F* = 2.581; df = 2,68; *p* = 0.083
Tinnitus awareness (0–100)	62.8 ± 30.1	50.0 ± 32.3	76.5 ± 140.8	*F* = 0.872; df = 2,70; *p* = 0.423
Tinnitus annoyance (0–100)	57.4 ± 32.9	49.1 ± 35.5	43.8 ± 31.0	*F* = 4.981; df = 2,68; *p* = 0.010
Tinnitus severity (0–5)	3.3 ± 1.1	2.7 ± 1.1	2.5 ± 1.1	*F* = 18.964; df = 2,68; *p* < 0.001
Tinnitus discomfort (0–10)	5.4 ± 3.0	4.4 ± 3.2	4.3 ± 3.2	*F* = 3.547; df = 2,68; *p* = 0.034
Tinnitus annoyance (0–10)	5.7 ± 3.2	4.1 ± 3.4	3.9 ± 3.2	*F* = 10.214; df = 2,68; *p* < 0.001
Tinnitus ignorability (0–10)	6.2 ± 3.4	4.8 ± 3.5	4.1 ± 3.2	*F* = 11.032; df = 2,68; *p* < 0.001
Tinnitus unpleasantness (0–10)	5.7 ± 3.1	4.3 ± 3.2	3.8 ± 3.2	*F* = 10.693; df = 2,68; *p* < 0.001
Mini tinnitus questionnaire (0-24; distress)	12.5 ± 6.5	10.3 ± 6.3	9.3 ± 6.3	*F* = 11.816; df = 2,66; *p* < 0.001
Tinnitus laterality (right/left/ left>right/right>left/ equal)	16/13/4/2/2	14/10/5/6/2	n.a.	χ^2^ = 34.585; *p* < 0.001
Tinnitus laterality (right/left/ left>right/right>left/ equal/inside head)	15/13/3/2/2	n.a.	9/11/4/4/5/2	χ^2^ = 40.558; *p* < 0.001
Tinnitus type (tone/noise/ crickets/other)	28/2/1/6	29/1/4/3	n.a.	χ^2^ = 17.196; *p* = 0.047
Tinnitus type (tone/noise/ crickets/other)	27/2/1/6	n.a.	29/3/1/3	χ^2^ = 17.200; *p* = 0.053
Tinnitus frequency (very high/high/middle/low)	5/17/10/5	9/16/9/3	n.a.	χ^2^ = 16.355; *p* = 0.016
Tinnitus frequency (very high/high/middle/low)	4/17/10/5	n.a.	5/20/8/3	χ^2^ = 18.147; *p* = 0.005
Mean hearing right (dB HL)	32 ± 32	26 ± 29	n.a.	*T* = 3.855; df = 33; *p* = 0.001
Mean hearing left (dB HL)	27 ± 25	23 ± 23	n.a.	*T* = 2.890; df = 33; *p* = 0.007

*Please note that for χ^2^ statistics statistical significance means that there is no change over time*.

## Discussion

### Discussion of Our Results

The aim of our study was to observe the transition from acute to chronic tinnitus and to identify predictors for the remission and trajectory of tinnitus. Our main results are (1) that among patients who present with a tinnitus duration of <4 weeks at the clinic only a small amount (18%) lose their tinnitus and show full remission, (2) that putative predictors for remission may be shorter tinnitus duration, female sex, lower consumption of alcoholic drinks, lower fear-related hyperacusis, and increased subjective ear pressure, and (3) that among those patients who develop chronic tinnitus, the perceptual tinnitus characteristics remain unchanged, whereas tinnitus distress improves over time.

To our knowledge, two studies with a comparable approach exist (14, 15). Particularly similar was the investigation by Wallhausser-Franke. Patients were also recruited between 2013 and 2016, the sample size and inclusion criteria (tinnitus onset below 4 weeks) were similar, the observation period equally was 6 months, and some rating instruments were identical. Our findings confirm the main findings of the two previous publications. Both studies indicate a high heterogeneity with respect to tinnitus etiology and applied treatments, both suggest remission rates below 20% and both converge in the observation that tinnitus characteristics (localization, sound quality) tend to stay stable, whereas the average tinnitus distress improves moderately over time. Predictor analysis was done with respect to variables of tinnitus distress as an outcome variable and not with respect to the complete remission of tinnitus. By this means, higher tinnitus distress and depressivity in the acute phase of tinnitus were identified as predictors for a worsening of tinnitus over time.

### Limitations

These three small studies highlight the need for further investigations of larger samples. The design of these investigations should consider the experiences and the limitations of the available studies. One lesson from previous undertakings is the need of a uniform definition of variables. For example, Wallhausser-Franke and colleagues find changes in tinnitus awareness over time that we weren't able to, which may be related to the operationalisation of this variable (15). Wallhausser-Franke coded this variable as “permanent” and “intermittent,” and we asked for percentage values (0–100%).

Another limitation of the available studies is the heterogeneity of performed interventions. These studies investigating the natural course of tinnitus over time should be complemented by interventional research. Even if we gathered data prospectively, it is not possible to draw causal conclusions as long as treatments are not controlled and randomized. For example, we can only speculate if a therapeutic intervention targeting the identified risk factors will improve the course of tinnitus. For example, consumption of alcoholic drinks was identified as one predictor. Only an interventional study could tell whether a corresponding intervention (e.g., decrease of alcohol consumption may help to increase the chance of remission or whether alcohol consumption is not a cause, but rather the consequence of a more affected chronic and decompensated tinnitus). Another limitation is the short period of a 6 months of follow-up and a missing question for thyroid disease.

### Perspective for Future Studies

In the future, increasing the validity of data by collecting a highly operationalized case history, interview, and diagnosis of patients as well as medical records, instead of relying solely on subjective reports as has been done in the present study, should be considered. In order to avoid a selection bias, one should aim not only to recruit from hospital clinics but also from ENT- and GP-practices. Moreover, one should aim at an early assessment of tinnitus patients as soon as possible after tinnitus onset. As heterogeneity of etiology and treatment is high, large-scale multi-center trials will be necessary to create a sufficiently large data basis.

The remission rate for acute tinnitus in our study is quite low. It is not clear whether the results are representative. One might assume a selection bias, as those patients in which the tinnitus did disappear spontaneously a few days after tinnitus onset did not present in the clinic. This selection bias is particularly relevant for those patients who present at a later timepoint after tinnitus onset. Accordingly, we observed that a longer tinnitus duration at presentation in the clinic was a predictor for a chronic manifestation of tinnitus.

The remission rate identified in our study and in comparable previous investigations (14, 15) was lower than remission rates for conditions such as sudden hearing loss and acoustic trauma [for overview see (15)]. Even if hearing recovery is a predictor for tinnitus improvement (6), our finding of a lower remission rate of tinnitus when compared to hearing recovery after sudden hearing loss suggests that the chances for hearing recovery after acute hearing loss are higher than the chances for tinnitus disappearance after acute tinnitus onset. Indeed, in clinical practice patients frequently report about good hearing recovery but ongoing tinnitus after sudden hearing loss.

These preliminary data will help in deducing hypotheses about which aspects might be relevant for the chronification of tinnitus. The finding that female sex is associated with a higher chance for tinnitus disappearance might explain why chronic tinnitus is more prevalent in men. Hyperacusis was speculated to be a precursor for tinnitus (9). It might be necessary to screen for hyperacusis in acute tinnitus patients and to find out which aspects of hyperacusis are relevant (in our sample this was fear-related, even if general anxiety did not predict remission). Interestingly, we did no see a significant effect of personality or anxiety even if there is evidence that these variables are involved in decompensated tinnitus (24, 25). It would be highly interesting to see whether ear pressure could be validated by means of diagnostic tests. One could speculate that the subjectively reported increased ear pressure might be an indicator of endolymphatic hydrops, which in turn might characterize a subtype of tinnitus with more fluctuations (26). For this purpose, a more exact definition of ear pressure is also necessary.

In conclusion, acute tinnitus is a highly heterogeneous phenomenon with up to 20% full recovery after 6 months and is taking a moderately positive trajectory in terms of tinnitus distress over time, despite unchanged tinnitus characteristics.

## Data Availability Statement

The raw data supporting the conclusions of this article will be made available by the authors, without undue reservation.

## Ethics Statement

The studies involving human participants were reviewed and approved by University of Regensburg, Germany. The patients/participants provided their written informed consent to participate in this study.

## Author Contributions

VV study concept, patient recruitment, manuscript, and statistics. RS patient recruitment. PK study concept and manuscript. BL study concept, patient recruitment, and manuscript. MS study concept, manuscript, and statistics. All authors contributed to the article and approved the submitted version.

## Conflict of Interest

The authors declare that the research was conducted in the absence of any commercial or financial relationships that could be construed as a potential conflict of interest.

## References

[1] Langguth B. Treatment of tinnitus. Curr Opin Otolaryngol Head Neck Surg. (2015) 23:361–8. 10.1097/MOO.0000000000000185 26261868

[2] Henry JA Reavis KM Griest SE Thielman EJ Theodoroff SM Grush LD. Tinnitus: an epidemiologic perspective. Otolaryngol Clin North Am. (2020) 53:481–99. 10.1016/j.otc.2020.03.002 32362561

[3] Maes IH Cima RF Vlaeyen JW Anteunis LJ Joore MA. Tinnitus: a cost study. Ear Hear. (2013) 34:508–14. 10.1097/AUD.0b013e31827d113a 23411656

[4] Schaette R Kempter R. Development of tinnitus-related neuronal hyperactivity through homeostatic plasticity after hearing loss: a computational model. Eur J Neurosci. (2006) 23:3124–38. 10.1111/j.1460-9568.2006.04774.x 16820003

[5] Gopinath B McMahon CM Rochtchina E Karpa MJ Mitchell P. Risk factors and impacts of incident tinnitus in older adults. Ann Epidemiol. (2010) 20:129–35. 10.1016/j.annepidem.2009.09.002 20123163

[6] Muhlmeier G Baguley D Cox T Suckfull M Meyer T. Characteristics and spontaneous recovery of tinnitus related to idiopathic sudden sensorineural hearing loss. Otol Neurotol. (2016) 37:634–41. 10.1097/MAO.0000000000001081 27228021 PMC4912237

[7] Sanchez TG Rocha CB. Diagnosis and management of somatosensory tinnitus: review article. Clinics. (2011) 66:1089–94. 10.1590/S1807-59322011000600028 21808880 PMC3129953

[8] Park E Kim H Choi IH Han HM Han K Jung HH. Psychiatric distress as a common risk factor for tinnitus and joint pain: a National Population-Based Survey. Clin Exp Otorhinolaryngol. (2019) 13:234–40. 10.21053/ceo.2019.00563 31842535 PMC7435435

[9] Schecklmann M Landgrebe M Langguth B T.R.I.Group DS. Phenotypic characteristics of hyperacusis in tinnitus. PLoS ONE. (2014) 9:e86944. 10.1371/journal.pone.0086944 24498000 PMC3908961

[10] Langguth B Kleinjung T Fischer B Hajak G Eichhammer P Sand PG. Tinnitus severity, depression, and the big five personality traits. Prog Brain Res. (2007) 166:221–5. 10.1016/S0079-6123(07)66020-8 17956786

[11] Holgers SZKM Svedlund K. Predictive factors for development of severe tinnitus suffering-further characterisation. Int J Audiol. (2005) 44:584–92. 10.1080/14992020500190235 16315449

[12] Wallhausser-Franke E Delb W Balkenhol T Hiller W Hormann K. Tinnitus-related distress and the personality characteristic resilience. Neural Plast. (2014) 2014:370307. 10.1155/2014/370307 25120934 PMC4121180

[13] D'Amelio CAR Scholz S Falkai P Plinkert PK Delb W. Psychological distress associated with acute tinnitus. HNO. (2004) 52:599–603. 10.1007/s00106-003-0944-5 15309256

[14] Olderog M Langenbach M Michel O Brusis T Kohle K. Predictors and mechanisms of tinnitus distress—a longitudinal analysis. Laryngorhinootologie. (2004) 83:5–13. 10.1055/s-2004-814235 14740299

[15] Wallhausser-Franke E D'Amelio R Glauner A Delb W Servais JJ Hormann K. Transition from acute to chronic tinnitus: predictors for the development of chronic distressing tinnitus. Front Neurol. (2017) 8:605. 10.3389/fneur.2017.00605 29209267 PMC5701924

[16] Forti S Ambrosetti U Crocetti A Del Bo L. Tinnitus patients lost to follow-up. Int J Audiol. (2010) 49:877–80. 10.3109/14992027.2010.505583 21070123

[17] Cima RFF Mazurek B Haider H Kikidis D Lapira A Norena A. A multidisciplinary European guideline for tinnitus: diagnostics, assessment, and treatment. HNO. (2019) 67:10–42. 10.1007/s00106-019-0633-7 30847513

[18] Landgrebe M Zeman F Koller M Eberl Y Mohr M Reiter J. The Tinnitus Research Initiative (TRI) database: a new approach for delineation of tinnitus subtypes and generation of predictors for treatment outcome. BMC Med Inform Decis Mak. (2010) 10:42. 10.1186/1472-6947-10-42 20682024 PMC2920857

[19] Manning C Grush L Thielman E Roberts L Henry JA. Comparison of tinnitus loudness measures: matching, rating, and scaling. Am J Audiol. (2019) 28:137–43. 10.1044/2018_AJA-17-0115 30938558

[20] Hesse G Tinnitus. Stuttgart, KG: Georg Thieme Verlag (2008).

[21] Rammstedt KCJB Klein MC Beierlein C Kovaleva A Eine kurze Skala zur Messung der fünf Dimensionen der Persönlichkeit: Big-Five-Inventory-10 (BFI-10); GESIS-Working Papers 2012|22. Köln/Mannheim: GESIS-Leibniz-Institut für Sozialwissenschaften (2012).

[22] Glaesmer HJH Klotsche J Herzberg PJ Deutsche Version der Revision des Life-Orientation-Tests (LOT-R). Heft: Zeitschrift für Gesundheitspsychologie (2008).

[23] Spitzer KMR Williams J Löwe B. A brief measure for assessing generalized anxiety disorder: the GAD-7. Archiv Internal Med. (2006) 166:1092–7. 10.1001/archinte.166.10.1092 16717171

[24] Fuller TE Cima RFF Van den Bussche E Vlaeyen JWS. The fear of tinnitus questionnaire: toward a reliable and valid means of assessing fear in adults with tinnitus. Ear Hear. (2019) 40:1467–77. 10.1097/AUD.0000000000000728 30998546

[25] Simoes J Schlee W Schecklmann M Langguth B Farahmand D Neff P. Big five personality traits are associated with tinnitus improvement over time. Sci Rep. (2019) 9:18234. 10.1038/s41598-019-53845-4 31796761 PMC6890781

[26] De Luca P Cassandro C Ralli M Gioacchini FM Turchetta R Orlando MP. Dietary restriction for the treatment of Meniere's disease. Transl Med UniSa. (2020) 22:5–9. 32523900 PMC7265917

